# Exploring “Talent” in Medical Education: A Scoping Review

**DOI:** 10.5334/pme.1859

**Published:** 2026-02-04

**Authors:** Rebecca Preyra, Sujata Mishra, Heba Khan, Shreya Saha, Armaanpreet Dhillon, Amy Keuhl, Sandra Monteiro, Elizabeth M. Wooster, Michael Gottlieb, Alexander Peever, Teresa M. Chan

**Affiliations:** 1 MD Candidate, University of Alberta, Faculty of Medicine and Dentistry, Canada; 2 McMaster University, Faculty of Health Sciences, Canada; 3 Kinesiology, University of Toronto, Faculty of Social Work, Canada; 4 PhD(c), School of Medicine, Toronto Metropolitan University, Canada; 5 Professor, Department of Emergency Medicine, Rush University Medical Center, United States; 6 McMaster University, School of Business, Canada; 7 Dean, School of Medicine and Vice-President Medical Affairs, Toronto Metropolitan University, Canada; 8 Adjunct Scientist, McMaster Education Research, Innovation, and Theory (MERIT), and Clinical Professor (part-time), Division of Emergency Medicine/Division of Education & Innovation, Department of Medicine, McMaster University, Canada

## Abstract

**Background:**

The term ‘talent’ appears in health professions education (HPE) but is variably defined and often conflated with performance proxies. Through a scoping review, the authors sought to map how ‘talent’ and related terms are used/defined in medical education across stages and use cases.

**Methods:**

A scoping review (Arksey-O’Malley; Levac; PRISMA-ScR) with descriptive mapping and content analysis of charted items was performed. The search was conducted across OVID-Medline, PubMed, Scopus, and Web of Science, focusing on studies related to talent in medical education from 1946 to May 20, 2024. The authors included not only the term talent but also broadened the review to include adjacent concepts, such as aptitude and giftedness. Two reviewers independently assessed titles, abstracts and full texts using predefined inclusion and exclusion criteria. A third reviewer resolved screening discrepancies. Relevant concepts were mapped for reporting, and a content analysis identified research gaps, trends, and patterns across global, regional and specialty contexts. The papers were tiered into two groups: Tier 1, directly mentioning the term talent; Tier 2, adjacent terms often related to talent.

**Findings:**

The authors reviewed 189 studies loosely related to talent in medical education: 47 (25%) were Tier 1 papers that directly mentioned talent, and 142 (75%) were Tier 2 (adjacent terms). The literature primarily originated from North America (41%, 77/189) and Europe (30%, 56/189) Most papers focused on identifying individuals with high potential (74%, 141/189), particularly in medical school selection, while less attention was given to themes like retention, equity and leadership.

**Conclusion:**

Although 47 papers contained the term “talent”, there was a paucity of papers that defined talent within medical education or applied a framework/theory. Interdisciplinary research may be a way to better introduce this concept to our field.

## Introduction

As healthcare systems grapple with physician burnout, workforce shortages, and aging populations, academic medical centers and medical education are confronting unprecedented challenges. In this context, traditional approaches to recruiting medical professionals are under increasing scrutiny. High turnover rates and attrition from primary care specialties have called into question whether we are identifying the right talent to meet the needs of the public, and if we are, how we might retain and foster such talent once they are in the system. This necessitates a closer examination of untapped human potential among both trainees and practicing physicians.

Medical educators must reevaluate how talent is conceptualized in medical education. Research in fields such as human resources (HR) and K-12 education emphazises systematic talent development, talent management, and retaining talented individuals through well-researched, structured approaches [1,2,3,4,5,6]. However, have such concepts entered into the zeitgeist of medical education or academic medical systems?

In contrast, talent management and retention in other fields (e.g. nursing, midwifery) have been shown to have an impact on organizational performance and patient outcomes [7,8]. Moreover, the concept of talent is discipline- or context-specific, which can confound its application in medicine, requiring tailored strategies to address its unique demands. As a field, medical education welcomes many groups of diverse backgrounds to engage in our literature [9,10].

### A Difference in Terminology

Interestingly, the term talent can be used differently depending on the originating literature. K-12 educators and education scientists tend to tease apart the concepts of giftedness (e.g. innate ability) and talent (the result of how a gifted individual interacts with training or education) [4,6,11]. For example, within HR-related papers in medicine and healthcare, the term “talent” may refer to any employee within an organization. In contrast, within the literature on selection and admissions in medicine, the term “talent” is related to individual differences in a person’s aptitude or abilities (e.g. visuospatial abilities).

Within medical education itself, the advent of movements such as competency-based medical education has shifted the scholarly conversation away from the term “talent” towards other constructs such as competence or capability [12,13,14,15,16]. Moreover, in efforts to align with the social accountability movement [17,18], diverse populations and changing population demographics emphasizes the need to integrate and embed principles of equity and diversity into medical education [19,20], fostering a medical workforce that reflects and effectively serves its communities – all the while not sacrificing excellence. All of this must be considered, of course, in light of generational challenges and shifting demographics of an aging physician corps [21]. And so, is it time now for us to think about how to introduce some of these other concepts of talent into our field? How might or present conceptualizations of talent interface with the work done in other disciplines or professions?

### How we conceptualize talent in medical education

As pressures continue to mount within both medicine and medical education to think more critically about the talented individuals in our profession, we must consider how the concept of talent has evolved over time and whether it meets the needs of our current context. For instance, what concept of talent might we apply when considering whether it is possible to sustain or enhance service delivery within the increasingly strained and reduced health workforce? Meanwhile, which definition or approach to evaluating talent should we use when selecting the right individuals for medical school or residency education?

Achieving these goals requires a deeper understanding of talent within the healthcare system. There is a need to define talent in medical education and develop strategies to nurture healthcare professionals who can thrive in high-pressure, complex environments. This study aimed to map the existing evidence and identify gaps in how talent is defined, identified, and managed in medical education.

We conducted a scoping review as they are particularly well-suited for exploring broad, emerging topics [22], highlighting underexplored areas, and establishing a foundation for more focused research—making them an ideal method for addressing the complexity and interdisciplinary nature of talent management in healthcare. We have opted not only to explore the concept of talent but also to examine adjacent terms (e.g., aptitude, clinical competence) that may reveal concepts that have been grouped with other movements in the literature. For instance, in the early 20^th^ century, there was a concerted effort to discern aptitude for medicine (e.g. prior to the development of standardized tests such as the Medical College Admissions Test). Later at the end of the 20^th^ century and in the beginning of the 21^st^ century, the competency-based movement ushered in a body of literature which we thought may have relevance to our research question.

## Methods

Our study followed the six-step Arksey and O’Malley framework for scoping reviews [23], enhanced by Levac et al.’s guidance for methodological rigour [24]. We adhered to the PRISMA-ScR reporting guideline to ensure transparency and reproducibility [25]. The detailed step-wise approach is outlined in the following paragraphs.

### Step 1: Identifying the Research Question(s)

Considering the recent pandemic-exacerbated changes to medical education and the practice of medicine, we observed a mass exodus of talented individuals from the field. This prompted curiosity around the operationalization and management of talent in medicine and medical education, leading to the following research questions: a) How has our field conducted scholarship to date to describe, justify, or clarify the current conceptualization of talent in medical education? and b) What definitions and frameworks have been used to date to directly describe the concept of talent in medical education?

### Step 2: Identifying Relevant Studies

After formulating the research question and developing key search terms, we consulted with a professional librarian to refine our search strategy and explore various databases. Our search covered four key databases (PubMed, OVID-Medline, Web of Science, and Scopus) and included literature from 1946 to May 20, 2024. The search strategy identified relevant literature on talent identification and aptitude in medical education and included terms and their variations, such as “talent*” OR “gifted” OR “aptitude*” OR “endowed student*”. Terms related to healthcare professions were incorporated via our pilot search to broaden our inclusion of possibly related fields, including “nurs*” OR “pharm*” OR “dentist*”. We sought to broaden the search’s catchment by including the term’ talent,’ as we acknowledge that there is terminological diversity in how various authors have written about the concept of talent. To capture literature specific to medical education, we included subject headings and keywords such as “education, medical”, “education, medical, graduate”, “education, medical, undergraduate”, “students, medical”, “internship and residency”, “clinical competence”, and “postgraduate education”. Boolean operators were applied to combine these terms, and both Medical Subject Headings (MeSH) and free-text terms were used to ensure a broad search. No search restrictions were placed on study date, location or design. We restricted our search to English-language studies, as the term talent and its selected synonyms were specific to English-language nuances and might not be directly generalizable to other languages.

Appendix A presents the search terms and strategy used in the OVID-Medline database that were then translated into the other database formats via our library’s online portal.

### Step 3: Study Selection

All studies matching the key terms were included for abstract and title screening using *Covidence* (Covidence, Australia). Prior to screening, reviewers (AD, AK, MG, RP, SM & SS) conducted a pilot screening of 10 studies to calibrate their understanding of the inclusion criteria and ensure consistency and reliability in study selection across the team. Title and abstract screening were performed independently by two reviewers, with each study assessed for inclusion in the full-text review stage. Full texts of studies marked for inclusion were retrieved, and two reviewers independently assessed each study’s eligibility for the final review. Discrepancies at the title and abstract or full-text stages were resolved through discussion between the two reviewers. If consensus could not be reached, a third reviewer was consulted to resolve the conflict.

This review focused on the definition and management of talent in medical education. We included studies conducted in medical education or a medical context regarding how talent is identified, recruited, managed, or developed. Eligible studies explored theories, frameworks, models of talent, and tools for evaluating medical aptitude, academic performance and systemic barriers to retention. The study population included medical school applicants, medical students, residents, physician learners, and educators. We considered studies in English and imposed no time period restrictions. We excluded studies unrelated to medical education, and those lacking full-text access.

### Step 4: Data Extraction

In alignment with the research question and drawing on the *Best Evidence in Medical Education* data extraction tool [26], we adapted a data extraction form from a previously published scoping review by our senior author [27]. The extraction tool was designed to incorporate elements of the PRISMA-ScR checklist, along with additional items based on the framework by Cook, Schmidt and Bordage for types of scholarship (description, justification, and clarification) [28]. We applied the Cook-Schmidt-Bordage typology only to empirical studies. Non-empirical conceptual pieces were enumerated separately. We also collected data on demographics, study type, geographic location, central specialties or populations examined, and the aspect of talent explored (e.g. identification, development, recruitment or management).

Once finalized, we deployed the extraction tool in Google Forms (Mountain View, CA) to facilitate data entry (see Appendix B for full tool). Following a calibration exercise with a randomly selected sample of 10 articles, relevant data from included studies were independently coded by reviewers (AD, AK, MG, RP, SM & SS).

### Step 5: Collating, Summarizing, and Reporting the Results

We employed a combination of descriptive summaries and content analysis to synthesize evidence for this scoping review. We also conducted content analyses to isolate recurring and overlapping categories across articles. Finally, we collated key facets common to all papers, including the geographical prevalence of talent research, specialties investigated, the top medical education journals publishing on this topic and the populations studied. For reporting, we present findings in two bands: 1) Tier 1: Core (‘talent’ explicitly used/defined) and 2) Tier 2: Adjacent (related performance terms). Percentages use the number of included records as the denominator unless otherwise specified. Records could map to multiple terms; therefore, term totals exceed 189.

### Step 6: Consultation Exercise

In the final step of Arksey and O’Malley’s framework [23] and in alignment with Levac’s 2010 publication [24], we engaged field experts and knowledge users to offer their insights on the completed research in an advisory capacity. We conducted an expert advisory step to sense-check the maps (three senior scholars across HPE/HRM/K-12). Advisors reviewed our preliminary tables and commented on face validity and gaps; the feedback informed interpretation wording only, not study selection or coding. These individuals participated in a series of expert mapping exercises around the use of talent in other fields. Additionally, we invited experts to suggest any further analyses that could be performed. We gathered insightful reflections that further refined our paper, such as the importance of acknowledging the diversity of approaches and disciplines within the medical education field and its impact on terminology variability. Advisors identified no additional eligible HPE frameworks. One suggested study appeared after our search cut-off and was therefore not included. Additionally, they suggested interpreting our findings in alignment with the medical education life cycle. Appendix C lists our expert consultants.

## Results

**Figure 1:**
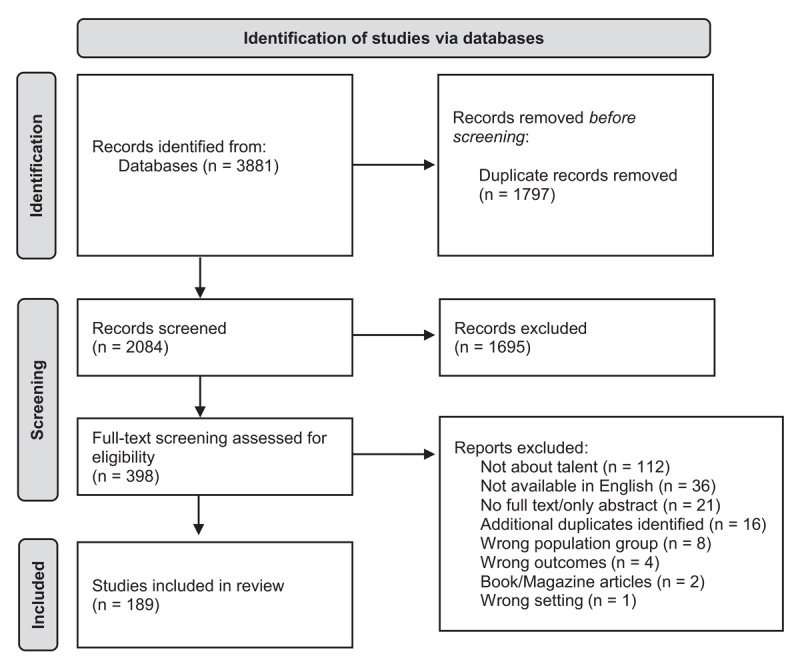
PRISMA flow diagram of study identification, screening, and inclusion.

Our search identified 3,881 potential citations. Following screening, 189 articles were selected for inclusion in the review (Figure 1). Appendix D provides a table sorting all 189 papers by talent or talent-related terminology.

The terms used in the 189 papers varied widely. Amongst these papers, there were 47 papers (24.6%) that were considered “Tier 1” papers that explicitly used the word talent (Appendix E). Tier 2 papers (i.e. other papers using the talent-adjacent terms from Table 1), such as skill(s) (n = 146, 76%) aptitude (n = 104, 54%), ability (n = 75, 39%), and competent/competence/competency (n = 50, 26%) (see Table 1 for a complete listing of the terms; while Appendix F is a glossary of the terms that we found within our search).

**Table 1:** Frequency of the term “talent” and related terms in the reviewed literature.


EXPANDED SEARCH TERM USED	FREQUENCY OF THE TERM IN OUR FINAL LIST OF PAPERS

**Core Definition (Tier 1)**	

Talent	47

**Adjacent terms (Tier 2)**	

Skills	146

Aptitude	104

Ability	75

Competen* (e.g. Competent/Competence/Competency)	50

Excellen* (e.g. Excellence/Excellent)	36

Performance	33

Success/Successful	14

Achievement or Attainment	8

Gifted	8

Intelligence	7

Expert/Expertise	3

Attributes	2

Knowledge	2

Accomplishment	1

Best	1

Grit	1

Proficiency	1

Qualified	1

Rock star	1


NB: The papers that we analyzed often used more than one term within their paper. As such, the total is more than 189. Also, see Appendix F for a glossary that attempts to define these terms. For reporting, we present findings in two bands: Core (‘talent’ explicitly used/defined) and Adjacent (related performance terms). This preserves breadth while distinguishing definitional specificity.”

**Figure 2:**
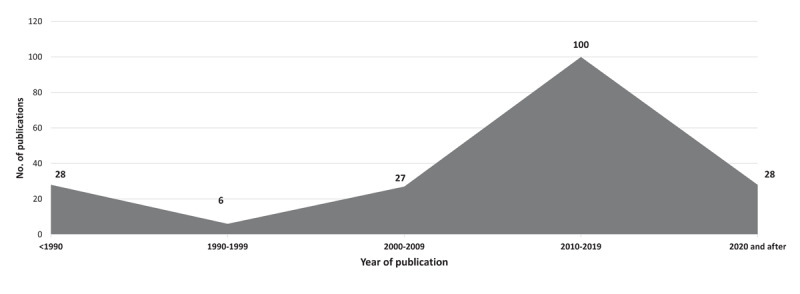
Number of papers using “talent” or related terms by year of publication.

Of the studies included in our review, 16% (31/189) did not reference a specific region. Among the remaining studies, the majority were conducted in North America (77/189, 41%) and Europe (56/189, 30%), with additional contributions from Asia (n = 10, 5%), Australia/New Zealand (11/189, 6%), South America (1/189, 0.5%), and multiple continents (4/189, 2%). Over the past two decades, publications featuring talent or related terms in medical education have increased, with more than half (100) of the included studies published between 2010–2019 (Figure 2).

### Types of Scholarship

Conceptual pieces were excluded in the Cook et al. framework, which focused on classifying empirical research. However, since we were exploring how our field uses talent-related terms, we chose to quantify this work. This decision was informed by our pilot review, which revealed the term “talent” was rarely used in empirical studies. We applied the Cook et al. framework to classify the empirical studies included in our review into three categories: Description, Justification, and Clarification. We separately examined field-advancing scholarship that we labeled as non-empirical conceptual papers, such as opinion pieces (e.g. commentaries, editorials, perspectives) or purely narrative reviews. Non-empirical conceptual papers comprised the second-largest category, accounting for 27% (51/189) of the included studies.

Description studies, which examined systems or phenomena, accounted for 13% (n = 25). Justification studies, which assessed the effectiveness of interventions, comprised 12% (n = 23).

### Methods used in the literature

With regards to the distribution of study methods across the reviewed literature, we found that quantitative studies comprised more than half of all the included studies (n = 110, 58%), followed by non-empirical studies (n = 56, 29.3%), qualitative studies (n = 10, 5.2%), systematic or literature reviews (n = 7, 3.7%), mixed methods (n = 6, 3.1%). Additionally, systematic reviews or meta-analyses constituted 4% (n = 7).

Among studies that discussed a specific study population group (n = 158, 83%), medical students or pre-clerkship learners were the most frequently represented group. Ninety-seven studies (51%) focused exclusively on this group, while 124 studies (65%) included them alongside other populations. Residents and fellows (practicing trainees) were the second most frequently discussed group, appearing exclusively in 33 studies (17.3%) and included in a total of 46 studies (24.1%). Medical school applicants were the primary study population in 16 studies (8.5%) and were included in a total of 19 studies (10%). In addition, 73 studies (38%) focused on a specific area of medical specialty. Surgical specialties were the most discussed, comprising 59% (n = 43) of this subset.

Content analysis of the included studies indicated that approximately 47% (n = 89) of the studies explored more than one area within the topic of talent. Identifying talent was the most frequently discussed topic (n = 137, 72%), followed by talent development (n = 43, 23%) and recruitment (n = 29, 15%). Less commonly addressed themes included retention (n = 16, 8%), talent management (n = 15, 8%), and the institutional culture around talent (n = 13, 7%). Topics such as emerging new leaders (n = 7, 4%), equity, diversity, and inclusion (n = 3, 2%), and defining talent (2%, n = 3) were rarely explored. The least discussed themes were emotional intelligence and multi-talented individuals (e.g., musician-physicians or Olympian-surgeons), each mentioned in only one study [29] (n = 1, 1%). A summary of the talent-related themes in the included literature can be found in Appendix G.

### Theories & Frameworks included

A total of 47 studies included a theory or framework that explored skills and competencies related to talent in medical education. However, only two studies provided an overarching framework of talent [30,31]. The remaining 45 studies were classified into three groups based on their focus on perceptual skills, technical skills or non-technical skills. Perceptual skills were defined as the capacity to recognize and interpret environmental cues, then integrate this information with existing motor abilities and knowledge to guide appropriate actions [32]. Seven studies (7/45, 15%) explored perceptual skills such as visuospatial abilities. Technical skills referred to the psychomotor abilities required to perform certain tasks, typically involving specific tools or procedures [33]. Eight studies (8/45, 18%) examined technical skills, including dexterity and psychomotor ability. Non-technical skills were defined as the personal and social abilities that enable individuals to interact effectively and safely within a team to manage complex situations [34]. Thirty studies (30/45, 67%) assessed non-technical skills, including cognitive and interpersonal abilities, such as communication, teamwork and leadership. Appendix H summarizes the frameworks for perceptual, technical and non-technical skills, respectively.

### Definitions of Talent

Although a total of 47 papers [30,31,35,36,37,38,39,40,41,42,43,44,45,46,47,48,49,50,51,52,53,54,55,56,57,58,59,60,61,62,63,64,65,66,67,68,69,70,71,72,73,74,75] mentioned the term talent. Only 7 of these papers attempted to define their use of the term ‘talent’. These definitions varied between the included articles. See Table 2 for definitions of talent in the literature [30,31,39,40,49,53,72].

**Table 2:** Definitions of talent in the literaturea.


FIRST AUTHOR, YEAR	TYPE OF TALENT DEFINED	DEFINITION USED

Bell, 2011	Personal Talents (of surgical training applicants)	Defined by the “TriMetrix Personal Talent Report”, an online survey that assesses behavioural style, intrinsic motivators, and dimensional balance.

Bell, 2012	Personal Talents (of surgical training applicants)	Defined by the second and third components of the TriMetrix assessment: intrinsic motivation and personal skills inventory.

Friedman, 2019	Talented physicians	“What has been shared in this review perhaps, is the paradigm of what it means to be talented: now, it is more than great book knowledge, bedside experience and technical expertise; it is the ability to recognize one’s strengths and vulnerabilities; to commit to practicing the highest quality and safest medicine with a willingness to adopt change to make care safer; to partner with colleagues to create a fair, respectful and equitable culture in the workplace and above all, to care for oneself and colleagues so that we can better care for our patients.” (p.93)

Jensen, 2017	Surgical talent	Three key elements for conceptualizing surgical talent: (1) Individual skills make the surgical prospect “good”, (2) a mixture of skills gives the surgical prospect the potential to become talented, and (3) becoming talented may rely on the fit between person and environment.

Kim, 2012	Medical students with exceptional talents in science and mathematics.	The “special admission group” consisted of students who received awards at national or international Olympiads in Korea.

Subramaniam, 2015	Talent development among trainee doctors	Talent development refers to the competency development of medical practitioners, geared towards producing competent professionals with the necessary skills for medical practice.

Wenzel, 2016	Latent talent among medical students or residents	“…a medical student, resident, or faculty colleague who will blossom academically or clinically” – given the right mentorship.


### Evolution of Terminology Used to Describe Talent in Medical Education

**Figure 3:**
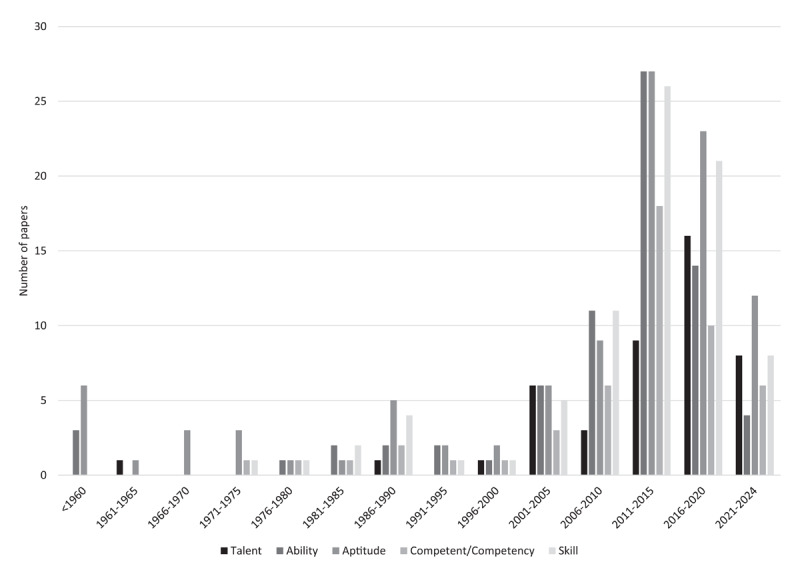
Talent and most common talent-adjacent terminology over the years.

The terminology used to describe talent, as reflected in adjacent terms in the literature, shifted over time (as shown in Figure 3). Prior to 1970, the literature predominantly used the term “aptitude” (9 papers [76,77,78,79,80,81,82,83,84]) and “ability” (3 papers [76,78,81]), with minimal reference to “talent” (1 papers [71]) and other related terms. From the 1970s to the 1990s, the term “aptitude” remained the most frequently used, while “ability” and “skill” appeared sporadically. The early 2000s exhibited a marked increase in the use of the term “talent” (6 papers [41,42,51,65,73,85] from 2001–2005), coinciding with a broader diversification of terminology, including more frequent mentions of “competency” or “competent” (3 studies from 2001–2005 [51,65,73]). Between 2011 and 2015, “aptitude” (28 papers [59,86,87,88,89,90,91,92,93,94,95,96,97,97,98,99,100,101,102,103,104,105,106,107,108,109,110]) and “ability” (27 papers [59,68,87,90,91,92,98,99,101,102,103,104,105,107,108,109,111,112,113,114,115,116,117,118,119,120,121]) peaked in usage, along with a marked increase in “skill” (26 papers [59,60,68,86,89,90,91,94,97,99,102,104,106,107,108,109,110,112,114,115,116,119,120,121,122,123]) and “competency” (18 papers [59,67,68,89,93,97,99,107,108,110,115,116,118,120,121,124]). More recent literature (2016–2024) shows a decline in “ability” and “aptitude”, while “talent” appeared more frequently, particularly in 2016–2020 (16 papers [30,35,43,48,49,50,54,55,57,61,62,63,72,75,125,126]).

### Consultation

Through our consultation with various experts in diverse fields, the most salient reflection noted was that medical education has shifted away from using the term ‘talent’ within the field and has largely been restricted to selection/admissions processes. Experts noted that there are numerous conceptualizations of talent from other fields that may apply to various aspects of the continuum of education through to training in the field (e.g. residency) to health human resources planning in the present physician/healthcare workforce. Our external consultations revealed no *additional* papers or frameworks from within medical education or health professions education worthy of inclusion. Our expert consultations also revealed one paper that we did not include in the review since it was published after May 20, 2024 [127].

## Discussion

This scoping review provides a broad analysis of research on talent in medical education, revealing progress but also significant gaps. Given the scoping design and absence of quality appraisal, we map what has been studied rather than evaluate effectiveness. Overall, our findings suggest several understudied areas (retention, talent management, equity) warrant targeted primary research and concept clarification.

The qualitative study from our expert consultation would have added only one more theoretical framework to this list of definitions of exceptional medical learners, rather than defining talent within the medical profession or other health professions [127]. This scoping review provided an overview of the complex landscape of research and scholarship, with implications for understanding, identifying, and nurturing talent among medical professionals.

Jensen et al. (2017) used a qualitative approach to identify three elements for conceptualizing surgical talent: (1) individual skill; (2) mix of skills that have the potential to *become* talented; (3) talent being a fit between the person and the environment [30]. Meanwhile, Subramaniam adopts a more human-resources oriented framework for speaking about talent development in their paper, where they highlight that structures that support talent development include coaching and mentoring individuals [31].

The geographical distribution of studies in this review indicated a strong concentration in North America and Europe, suggesting a potential bias in the current understanding of talent in medical education, which may have been an artefact of our language restriction to English. The absence of studies from Africa and the limited representation from South America and Asia pointed to a dominant Western-centric view of talent in medical education. Meanwhile, we also noted that over 15% of the papers did not mention a specific location of origin.

The types of scholarship identified suggested that the conceptualization of talent in medical education remains an evolving field. Clarification and conceptual pieces make the majority of types of scholarship in the included papers. This illustrates a focus on theoretical development, testing, evaluation and debate. This shift is important; however, it may also indicate overinflated reporting, as we included non-empirical studies in our classification of types of scholarship. These findings pointed to both the complexity of defining talent in medical education and the need for continued exploration to refine and apply this concept effectively.

The methodological approaches in the included studies demonstrated a strong preference for quantitative methods, reflecting a focus on hypothesis-driven research consistent with the traditional biomedical research paradigm. While valuable, this emphasis limited opportunities for exploratory or building approaches. Qualitative studies remain underutilized, potentially creating a gap, since this type of investigation may better shed light on the nuanced and contextual dimensions of talent in medical education.

Our review highlighted a strong emphasis on how the field identifies talent. This aligns with traditional medical education practices, where talent is often equated with academic achievements such as high-test scores and grades [128,129]. The implications for our are that conversations that have occurred in other fields (such as K-12 education) around equity in talent identification within selection processes are absent due to the nascent nature of the literature base. A substantial number of studies concentrated on pre-medical, medical students, and pre-clerkship learners, reflecting the priority placed on early identification and development of talent within our learner populations. This aligned with conventional views of giftedness as an innate quality to be recognized early. However, such an approach risked neglecting the ongoing development of talent throughout a physician’s career. The relatively low representation of studies addressing residents, fellows, and practicing physicians highlighted the need for future research on how talent evolves across different stages of medical careers.

### Going Beyond Medicine

During our expert consultation process, many of those consulted noted that adjacent fields, such as healthcare leadership or human resources, may contain insights, suggesting that an interdisciplinary or interprofessional approach may be warranted. This would require a much broader scoping review, as we had decided to restrict our search to medical education. In the future, it would be prudent to explore our present findings in relation to the literature in fields beyond medicine, healthcare, medical/health professions education.

Retention and talent management in medicine and medical education —critical for sustaining the healthcare workforce —were addressed in only 8% of studies. This is a significant gap given the challenges of physician burnout and workforce attrition. While identifying and recruiting talent are important, retaining and supporting healthcare professionals throughout their careers is equally vital. Research from organizational psychology reiterates the importance of fostering positive workplace environments to enhance retention [130,131], yet this perspective is largely absent from medical education research [132]. Similarly, attributes like emotional intelligence [133], adaptability/cognitive flexibility [134,135], leadership talent [136,137,138,139] —essential for modern healthcare—may be underexplored in medical education [138,140].

This raised important questions about why the talents of individuals within medicine and medical education are not more fully examined or supported. One possible explanation, based on the work by Ashforth & Schinoff on individual identity construction within organizations, is that medical education sets the bar of entry for the most talented individuals and then, through the process of medical education shifts their identity from uniquely talented individuals to a standardized doctor identity [141]. Thus, the highly competitive selection processes for medical training aim to recruit the most talented individuals, yet these talents are often merely reduced to considering competency after individuals undergo the process of professionalization [141]. This paper aimed to shift the focus toward empowering talented individuals to realize their full potential and addressing systemic barriers that hinder this shift. The concept of talent was deemed related to competency for the purpose of this review (for instance, in those who seem to achieve competency especially quickly); however, we do not feel that it is prudent to restrict our conceptualization of individuals to only their achievement of competency without regard for other conceptualizations of talent.

Analysis of the terminology (Figure 3) used in the literature to describe talent revealed significant shifts in the conceptualization of talent in medical education. Early discourse (pre-1970s) primarily framed talent in terms of “aptitude”. As medical education evolved, particularly in the late 20th century and early 21st century, there was a growing emphasis on skill acquisition and competency-based medical education, as evidenced by the rise in the use of terms such as “skill” and “competent”/“competency” from the 1980s onward. The increased use of the terms “competent” and “competency” to describe talent beginning in the 1980s with a marked increase in the early 2000s corresponds with an increase in competency-based medical education, particularly following the adoption of the CanMEDS framework (Canadian Medical Education Directives for Specialists) in 1996 [142]. and the Outcome Project of the ACGME (Accreditation Council for Graduate Medical Education in the USA) in 1998 [143]. From 2011–2015, there was a peak in the use of the terms “aptitude” and “ability”, which highlighted the continued use of established terminology in the discourse on medical education. However, the simultaneous increase in the terms “talent” and “competency” suggested a linguistic shift toward more diverse and nuanced ways of describing talent in medical education. This linguistic shift underscored the evolving understanding of talent in medical education, moving from a fixed concept of innate giftedness to a more dynamic, context-dependent view, similar to that described by the *Evolving Complexity Theory of Talent Development* by Dai, which originates within the field of K-12 education [6].

In recent years, concepts such as “competence” and “maintenance” of competency or certification dominated the medical education literature [133,144,145,146,147,148,149,150].Competency-based medical education (CBME) assesses learning by determining whether a learner can meet a predetermined standard [145]. The verbiage around the task of teaching physicians has regressed to a very pragmatic and protectionist view of ensuring graduates of medical training are competent, not talented or excellent [151]. Standardization of competence rather than talent may be driven by a moral imperative to protect patients, as the focus on being a professional supersedes being exceptional.

Ambitious, talented learners are thus discouraged from exploring and challenging themselves as they rush to achieve competence, creating a system in which all professionals achieve an average standard in their skills and practice, which may come at the expense of creativity and innovation. Within this framework, patient safety is promoted by ensuring that all physicians have a minimum level of competence. However, shifting our focus to produce highly talented and skilled physicians may inspire them to be more creative and pursue excellence, which, in turn, will improve patient safety in other ways.

Talent is inherently contextual, yet few studies in our review adequately operationalized or defined it as other fields have [6]. For instance, one study by Kim et al. in 2012 described talent narrowly, referring to Olympiad winners, while others used terminology suggesting exceptionalism without explicit definitions [53]. The lack of contextualized and nuanced descriptions of talent reflects a broader gap in medical education, where talent is not effectively leveraged to address workforce challenges. In contrast, the abundance of CBME literature demonstrated a disparity in attention, suggesting a lack of emphasis on fostering and utilizing talent [14]. Critically, we also observe that the bulk of the empirical literature does seem to originate from either North America or UK/European settings, which may privilege certain perspectives about talent; going forward it would be important for scientists from around the world to be invited to this conversation around talent to ensure we have a more comprehensive view of what this term may mean in diverse contexts.

Although talent is a well-established construct in other fields (e.g. human resources, organizational development, or even K-12 education), its underdeveloped discourse in medical education is problematic. At a time when healthcare systems face workforce shortages and rising demands, there is a pressing need to explore how talent intersects with more efficient training, better identification within clinical environments, and effective management of existing talent. Without renewed scholarly attention to this topic, the field risks missing innovative approaches to addressing human resource challenges in healthcare.

Some innovations and pilots have shown that a talent-based approach can serve as a powerful tool to strengthen the healthcare system and support the development of inherently gifted or exceptional individuals [152,153]. This aligns well with the concept of a strengths-based approach to education [154,155,156], which has some roots in indigenous teachings from Turtle Island [157]. Reimagining and contextualizing talent for modern medical education can help unlock the potential of trainees, physicians, and surgeons, ultimately benefiting patients and the healthcare system. We feel that it may be highly beneficial to occasionally shift the conversation from meeting minimum competencies to fostering exceptionalism and excellence in the healthcare workforce.

### Limitations

This study is not without its limitations. Firstly, we did not conduct an exhaustive literature search across all databases. The inclusion of interdisciplinary databases such as Web of Science, Scopus, and OVID-Medline, coupled with a broad search strategy, provided us with a large evidence base to capture relevant information. However, in our particular search, we note that heterogeneity in the terms used across the fields of medical education and health professions education may have led to under-identification of papers. Second, only studies in the English language were included in the review. Thirdly, we note a strong tie between the terms we found in the literature and our expanded synonym terms, which is unsurprising but certainly worth mentioning, as it affects the reader’s interpretation of both Table 1 and Appendix F. The Adjacent band includes broad performance terms; we attempted to mitigate construct drift by presenting Core vs Adjacent analyses. Finally, we did not include an assessment of the quality or rigor of included studies, which may limit our ability to evaluate the quality and strength of the evidence.

## Conclusion

This scoping review highlighted the complexity of talent in medical education and identified significant opportunities for further exploration. The findings emphasized the need for a more inclusive and comprehensive understanding of talent that moves beyond traditional academic metrics to encompass the diverse competencies required for excellence in healthcare. While current research has predominantly focused on talent identification, particularly among those seeking to become medical students, there has been limited attention to talent development, retention, and the dynamic nature of talent throughout a medical professional’s career.

The underrepresentation of themes such as equity, diversity, and inclusion pointed to a critical gap in the literature. Addressing this gap is essential to aligning talent frameworks with the goals of creating a diverse and equitable healthcare workforce capable of meeting the needs of varied patient populations. As healthcare systems evolve, so too must our understanding of talent, incorporating competency-based education and holistic approaches that recognize the broad spectrum of skills and experiences vital to success in medicine.

## Additional File

The additional file for this article can be found as follows:

10.5334/pme.1859.s1 Appendices. Appendix A to H.
