# Supplementary material for: Exploring “Talent” in Medical Education: A Scoping Review
Source: Perspect Med Educ. 2026 Feb 4;15(1):75–92. doi: 10.5334/pme.1859 (PMC12879997; doi:10.5334/pme.1859)
Supplement: Appendices. — Appendix A to H. [file pme-15-1-1859-s1.zip › pme-15-1-1859-s1/Appendix_A.docx]

**Appendix A**

**OVID Medline Database Key Search Terms**

Database: OVID Medline Epub Ahead of Print, In-Process & Other Non-Indexed Citations, Ovid MEDLINE(R) Daily and Ovid

MEDLINE(R) 1946 to 2024

Search Strategy:

| 1 | talent*.mp. | 7320 |
| --- | --- | --- |
| 2 | gifted.mp. | 1569 |
| 3 | aptitude?.mp. | 9552 |
| 4 | Aptitude/ | 4504 |
| 5 | endowed student?.mp. | 0 |
| 6 | nurs*.mp. | 833469 |
| 7 | pharm*.mp. | 4449540 |
| 8 | Dentist?.mp. | 61111 |
| 9 | 1 or 2 or 3 or 4 or 5 | 17576 |
| 10 | 6 or 7 or 8 | 5315754 |
| 11 | 9 not 10 | 16010 |
| 12 | education, medical/ or education, medical, graduate/ or "internship and residency"/ or education, medical, undergraduate/ | 165471 |
| 13 | Students, Medical/ | 45509 |
| 14 | Internship and Residency/ or Clinical Competence/ or postgraduate.mp. or Education, Medical, Graduate/ | 186880 |
| 15 | 12 or 13 or 14 | 277711 |
| 16 | 11 and 15 | 1051 |

This strategy was then replicated across the other databases.
