# Supplementary material for: Exploring “Talent” in Medical Education: A Scoping Review
Source: Perspect Med Educ. 2026 Feb 4;15(1):75–92. doi: 10.5334/pme.1859 (PMC12879997; doi:10.5334/pme.1859)
Supplement: Appendices. — Appendix A to H. [file pme-15-1-1859-s1.zip › pme-15-1-1859-s1/Appendix_B.pdf]

Talent in MedEd Scoping Review Extraction Tool

* Indicates required question

1. *

Mark only one oval.

- ( ) Teresa Chan
- ( ) Heba Khan
- ( ) Alex Peever

2. ID number of the paper *

____

3. Year of Publication *

____

4. Last name of first author: *

____

5. Does this paper meet our finalized inclusion criteria? *

Is this paper about talent, high performance, excellence of a medical professional (med student, resident, fellow, practicing physician)?

Mark only one oval.

- Yes
- No
- Maybe

6. If you answered No or Maybe in 5a., what was your hesitation?

Language Screen

7. Is this paper in English? *

Mark only one oval.

- Yes
- No (The Principal Investigator will review these)
- No, but you are Principal Investigator doing the review

Data Extraction

8. What type of scholarship was this? *

For full description of these types, check out this paper: https://onlinelibrary.wiley.com/doi/abs/10.1111/j.1365-2923.2007.02974.x For paper that are possibly both, please use the OTHER option and type the two types in.

Mark only one oval.

- Conceptual piece (i.e. narrative review, opinion, editorial, or other theory advancing commentary) *Skip to question 19*
- DESCRIPTION STUDY (of systems): e.g. we built a MD+ program program for talented individuals
- DESCRIPTION STUDY (usually of a phenomenon): focuses on the first step in the scientific method, namely, observation. e.g. Intervention descriptions outcome data reported, usually no comparisons.
- JUSTIFICATION STUDY: focuses on the last step in the scientific method by comparing one educational intervention with anot her to address the question(often implied): "Does the new intervention work?"
- CLARIFICATION STUDY: employ each step in the scientific method, starting with observations (typically building on prior research) and model s or theories, making predictions, and testing these predictions.
- Other: ____________________

9. Was there a specific specialty featured? If so, what?

e.g. Urology, Emergency Medicine

____

10. What types of population were most discussed within this paper? *

Check all that apply. e.g. If they are describing, discussing, or highlighting a certain population.

Check all that apply.

- [ ] Unknown
- [ ] Medical Students NOS - i.e. Level NOT specified
- [ ] Pre-clerkship Med Students
- [ ] Clerkship Med Students
- [ ] Residents/Fellows (Trainees who are practicing)
- [ ] Attending Physicians (Practicing qualified physicians)
- [ ] Basic or Non-Clinician Scientists
- [ ] Nurses
- [ ] Administrators (e.g. healthcare leaders w/o professional identity)
- [ ] Other healthprofessionals (OT, PT, Paramedics, Midwives - please clarify in "Other")
- [ ] Other: ______________________

Data Extraction re: Studies or Innovation Reports

11. Focus of Interest: *

Mark only one oval.

- ( ) Groups of People (Trainee, Clinician, etc..)
- ( ) Health System / Program
- ( ) Other: ______________________

12. Population Size (Sample Size):

Please describe the item of interest AND additional numbers etc. If you can write SMALL SCALE (Local study), MEDIUM SCALE (multi-centre), LARGE SCALE (national)

____

13. Timespan of DATA collection

____

14. In which continent(s) was study performed (select all that apply)? *

Check all that apply.

- [ ] N/A (e.g. Internet-based, commentary)
- [ ] Africa
- [ ] Asia
- [ ] Middle East
- [ ] Australia/New Zealand
- [ ] Europe
- [ ] North America
- [ ] South America
- [ ] Other: ____________________

15. What part of Talent relations did this paper focus upon? *

Check all that apply.

Check all that apply.

- [ ] Identifying Talent
- [ ] Recruitment
- [ ] Retention
- [ ] Talent Development
- [ ] Talent Management
- [ ] Emerging New Leaders
- [ ] Culture of the institution around Talent
- [ ] Multi-talented individuals (musician-physicians; olympian-surgeons)
- [ ] Other: ____________________

16. Theme *

What was/were the major theme(s) of the article? (Check all that apply)

Check all that apply.

- [ ] Finding talent
- [ ] Selecting talent
- [ ] Talent Management / Developing Talent
- [ ] Retention of Talent
- [ ] Suppression of talent
- [ ] Equity, diversity, and Inclusion
- [ ] Outcomes of having talented people to the ORGANIZATION
- [ ] Outcomes FOR talented people in education
- [ ] "Community of Practice" or other synonyms (cohorting talent, etc..)
- [ ] Other: ______________________

17. What data sources were used? *

Click N/A if it doesn't apply.

Check all that apply.

- [ ] N/A
- [ ] Substantive written texts (narratives, reflections, blog posts)
- [ ] Papers (literature review)
- [ ] Surveys (quantitative - number-based, questions Likert scales, other scales)
- [ ] Surveys (qualitative - open ended questions)
- [ ] Objective observations/Tests
- [ ] Interviews
- [ ] Focus Groups
- [ ] Ethnographic approaches (e.g. Observations with field notes, online observation)
- [ ] Simulation (e.g. Time to completion)
- [ ] Other: ______________________

18. Methodology *

For innovation reports, look at the results section of the program evaluation to determine what methods were used. If none were reported (i.e. innovation report without evaluation, then click the 4th option below to indicate it was a Descriptive Innovation Report only).

Mark only one oval.

- ◯ Quantitative *Skip to question 25*
- ◯ Qualitaitve *Skip to question 23*
- ◯ Mixed methods *Skip to question 23*
- ◯ Descriptive program report ONLY with no actual outcomes data. *Skip to question 28*
- ◯ Other: ____________

Data Extraction for Conceptual/Commentary Pieces

19. Focus of Interest: *

Mark only one oval.

- ◯ People (Trainee, Clinician, etc..)
- ◯ Program
- ◯ Other: ____________

20. Regarding which continent(s) was piece about (select all that apply)? *

Check all that apply.

- [ ] N/A (e.g. Internet-based, commentary)
- [ ] Africa
- [ ] Asia
- [ ] Middle East
- [ ] Australia
- [ ] Europe
- [ ] North America
- [ ] South America
- [ ] Other: ______________________

21. What part of Talent relations did this paper focus upon? *

Check all that apply.

Check all that apply.

- [ ] Identifying Talent
- [ ] Recruitment
- [ ] Retention
- [ ] Talent Development
- [ ] Talent Management
- [ ] Emerging New Leaders
- [ ] Culture of the institution around Talent
- [ ] Multi-talented individuals (musician-physicians; olympian-surgeons)
- [ ] Other: ______________________

22. What data sources were used? *

Click N/A if this does not apply.

Check all that apply.

- [ ] N/A
- [ ] Substantive written texts (narratives, reflections, blog posts)
- [ ] Papers (literature review)
- [ ] Surveys (quantitative - number-based, questions Likert scales, other scales)
- [ ] Surveys (qualitative - open ended questions)
- [ ] Objective observations/Tests
- [ ] Interviews
- [ ] Focus Groups
- [ ] Ethnographic approaches (e.g. Observations with field notes, online observation)
- [ ] Other: ______________________

Skip to question 28

Qualitative

23. What was the specific qualitative study design? *

Use what author says; add notes in "Other" if questions or disagree with authors

Check all that apply.

- [ ] Case Study
- [ ] Discourse analysis
- [ ] Ethnography (including online ethnography)
- [ ] Grounded theory
- [ ] Phenomenology
- [ ] Thematic analysis
- [ ] Program Evaluation - Survey free text
- [ ] Program Evaluation - interviews, focus groups
- [ ] Other: ______________________

24. Did you review a mixed methods paper? *

Mark only one oval.

- Yes *Skip to question 25*
- No *Skip to question 28*

Quantitative Method

25. What was the specific quantitative study design? Select all that apply. *

(Use what author says; add notes in "Other" if questions or disagree with authors).

Check all that apply.

- [ ] Descriptive
- [ ] Inferential (including psychometrics)
- [ ] Retrospective
- [ ] Prospective
- [ ] Program Evaluation
- [ ] Other: ______________________

26. Was this study an EXPERIMENT? *

i.e. in the methods do they specifically described as a RCT, or an experiment where they intervened at least one part of the population? (E.g. gave people access to a twitter account?)

Mark only one oval.

- Yes *Skip to question 27*
- No *Skip to question 28*
- Unsure *Skip to question 27*

Skip to question 28

Experimental Methods

27. What was the specific experimental study design? Select all that apply. (Use what author says; add notes in “Other” if questions or disagree with authors).

Check all that apply.

- [ ] Randomized controlled trial
- [ ] Pre-test / Post-test
- [ ] Single group, no comparison
- [ ] Single group, repeated measures
- [ ] Other: ______________________

Final questions

28. Anything interesting or curious about this paper?

____
____
____
____
____

29. Were you unsure about this paper in any way?

____
____
____
____
____

30. Is this article a potential candidate for discussion as an exemplar? *

Exemplar papers are outstanding examples that illustrate some key facet (E.g. it is a great descriptive paper).

Mark only one oval.

- Yes
- No
- Maybe

31. If yes, explain why?

____

32. Should this article be EXCLUDED from the review? *

e.g. too focused on non-MD populations, too old??

Mark only one oval.

- No
- Maybe
- Yes

33. If yes/maybe, explain why?

____

This content is neither created nor endorsed by Google.

Google Forms
