# Supplementary material for: Exploring “Talent” in Medical Education: A Scoping Review
Source: Perspect Med Educ. 2026 Feb 4;15(1):75–92. doi: 10.5334/pme.1859 (PMC12879997; doi:10.5334/pme.1859)
Supplement: Appendices. — Appendix A to H. [file pme-15-1-1859-s1.zip › pme-15-1-1859-s1/Appendix_C.docx]

**Appendix C: Experts**

| **Name** | **Position** |
| --- | --- |
| Ajiri Ikede | Military Physician, Department of National Defense  Staff Officer Retention, Canadian Forces Health Services Headquarters, Ottawa, Canada (28 years in the Canadian Armed Forces) |
| Andrew Downes | Major General (Retired), Former Surgeon General and Commander of the Canadian Forces Health Services Group, Ottawa, Canada (31 years in the Canadian Armed Forces) |
| Ann Koritko | Retired Principal, Halton Catholic District School Board, various schools, Canada  Teacher (23 years), Administrator (12 years) |
| Fei Song | Professor and Graduate Program Director, MScM and PhD programs Ted Rogers School of Management, Toronto Metropolitan University, Canada (20 years) |
| Kelly Kimens | Executive Vice President, People and Governance, William Osler Health System, Brampton, Canada |
| Kevin Sebastian | Principal, Balmoral Drive Sr. Public School, Peel District School Board, Canada  Teacher (14 years), Administrator (11 years) |
| Maggie Thornton | Margaret Thornton, Assistant Professor, Educational Leadership, Administration, and Research, Rowan University’s College of Education, New Jersey, United States (7 years research experience) |
| Rakesh Patel | Professor of Medical Education and Head of MBBS (2 years)Deputy Director, Institute of Health Sciences Education, Faculty of Medicine and Dentistry, Queen Mary University of London, United Kingdom Badminton and hockey player (6 years) |
| Robert Jay | Clinical Associate Professor and Director of Clinical Skills, University of Lincoln, United Kingdom (2 years) Qualified swimming teacher, former cricket Team Captain, and Safeguarding Officer and Club Chairman for Electricity Sports Cricket Club Leicester, United Kingdom |
| Shalini Phillips | CEO, The Fractional CHRO, Mississauga, Canada (over 20 years in HR) |
| Wendy Coates | Professor of Emergency Medicine, Education Scientist, Dance Medicine Specialist, Department of Emergency Medicine, Los Angeles David Geffen School of Medicine, University of California, United States  Former professional ballet dancer (10 years) |
| Zach Smith | Staff Sergeant, United States Army (6 years)  Stanford Emergency Medicine, PGY-4, Stanford University School of Medicine, United States |
