# Supplementary material for: Exploring “Talent” in Medical Education: A Scoping Review
Source: Perspect Med Educ. 2026 Feb 4;15(1):75–92. doi: 10.5334/pme.1859 (PMC12879997; doi:10.5334/pme.1859)
Supplement: Appendices. — Appendix A to H. [file pme-15-1-1859-s1.zip › pme-15-1-1859-s1/Appendix_D.docx]

**Appendix E: Summary of Publications by Talent-Related Terminology**

| **Term** | **Citation** |
| --- | --- |
| Talent | Abdulrahman, 2016; Ackermann, 2023; Aggarwal, 2015; Alexander, 1988; Bell, 2011; Bell, 2012; Boudoulas, 2005; Carnes, 2001; Cobb, 2020; Deuchler, 2022; Dhaliwal, 2021; Durso, 2009; Engel-Rebitzer, 2023; Findeklee, 2019; Friedman, 2018; Hardre, 2016; Hayden, 2005; Jensen, 2017; Khawar, 2022; Kim, 2012; Kramp, 2016; Lindlohr, 2017; Lucey, 2010; Lucey, 2020; Meier, 2016; Moglia, 2014; Nothnagle, 2014; Pfeifer, 2017; Porter, 2017; Preece, 2018; Rathmell, 2023; Roman, 2004; Rosenthal, 2006; Sadideen, 2013; Satiani, 2014; Sternberg, 2008; Subramaniam, 2015; Sun, 2021; Sutton, 2018; Tucker, 1965; Wenzel, 2016; White, 2002; Woodrow, 2022; Zuckerman, 2018 |
| Ability | Azari, 2019; Brannick, 2009; Buckley, 2014; Burkhart, 2014; Engel-Rebitzer, 2023; Gallagher, 2009; Garrud, 2018; Gliatto, 2016; Groenier, 2014; Hale, 1946; Harrington, 2018; Harris, 2015; Hassan, 2007; Hayden, 2005; Helle, 2010; Henry, 1990; Hewer, 1956; Koczwara, 2012; Koenig, 2013; Kramp, 2016; Kulasegaram, 2013; Lambe, 2012; Langlois, 2015; Leff, 2008; Leinster, 2013; Krespi, 1986; Libbrecht, 2014; Lievens, 2012; Lindlohr, 2017; Louridas, 2016; Luursema, 2010; Maan, 2012; Macmillan, 1999; Maharjan, 2022; Mahon, 2013; Malhotra, 2015; Mason, 2021; Matarazzo, 1972; Mathers, 2016; McClusky , 2005; McManus, 2003; McManus, 2013; Mendis, 2011; Meyer, 2019; Migliaretti, 2017; Moglia, 2014; Moore, 2015; Muller , 2010; Nayar, 2020; O'Donovan, 2012; Oliver, 2012; Poole, 2012; Pounds, 2017; Quiillin, 2013; Robb, 2007; Roman, 2004; Rosenthal, 2006; Sapira, 1979; Sartania, 2014; Satiani, 2014; Schreurs, 2020; Šimunovic, 2004; Singh, 2017; Siska, 2015; Smoker, 1984; Sobral, 1995; Stefanidis, 2006; Sternberg, 2008; Stuit, 1948; Sun, 2021; Watson, 2017; White, 2002; Wright, 2010; Xu, 1993; Yates, 2013 |
| Accomplishment | Hale, 1946 |
| Achievement | Collin, 2009; Glaser, 1954; Hecker, 2009; Lambe, 2012; McManus, 2003; Rothman, 1973 |
| Aptitude | Azari, 2019; Beattie, 2022; Bech, 2013; Bowden, 2023; Buckley, 2013; Buckley, 2014; Bulstrode, 2003; Byrne, 1971; Carr, 2009; Cerutti, 2013; Collin, 2009; Deuchler, 2022; ElBoghdady, 2021; Glaser, 1954; Gliatto, 2016; Gough, 1967; Griffin, 2021; Groenier, 2014; Hale, 1946; Harrington, 2018; Harris, 2015; Hassan, 2007; Hecker, 2009; Helle, 2010; Henry, 1990; Hewer, 1956; Holmes, 1988; Hunter, 1965; Husbands, 2013; Husbands, 2014; Husbands, 2014; Jensen, 2017; Korets, 2011; Kramp, 2016; Krupat, 2017; Kulasegaram, 2013; Kulkarni, 2022; Kumwenda, 2018; Lambe, 2012; Leff, 2008; Leinster, 2013; Krespi, 1986; Lindlohr, 2017; Lipton, 1988; Louridas, 2016; Lucey, 2020; Maan, 2012; Macmillan, 1999; Maharjan, 2022; Malhotra, 2015; Mason, 2021; Mathers, 2016; Mathew, 2018; McClusky , 2005; McGaghie, 2002; McManus, 2003; McManus, 2013; McManus, 2013; McManus, 2013; Mendis, 2011; Mercer, 2018; Meyer, 2019; Moglia, 2014; Moore, 2015; Nayar, 2020; Osborn, 2015; Phillips, 2018; Poole, 2012; Pounds, 2017; Van Rij, 1995; Roman, 2004; Roth, 1996; Sapira, 1979; Sartania, 2014; Schofield, 1966; Schreurs, 2020; Schropel, 2024; Shulruf, 2020; Šimunovic, 1975; Singh, 2017; Sladek, 2016; Sternberg, 2008; Stuit, 1948; Sulkoff, 1968; Sun, 2022; Sutton, 2018; Swanstrom, 2006; Tang, 2014; Toale, 2024; Vaughn, 1946; Vu, 1987; Waheed, 2011; Wanzel, 2002; Watson, 2017; Watson, 1955; Weinstein, 1974; West, 2011; White, 2002; Wilson, 2012; Woodrow, 2022; Wright, 2010; Xu, 1993; Yates, 2013; Yates, 2010 |
| Attainment | McManus, 2013 |
| Attributes | Adam, 2015 |
| Attitude | Sapira, 1979 |
| Competency/Competent | Collin, 2009; Engel-Rebitzer, 2023; Gallagher, 2009; Garrud, 2018; Harrington, 2018; Harris, 2015; Hayden, 2005; Hecker, 2009; Holmes, 1988; Husbands, 2013; Koenig, 2013; Kulkarni, 2022; Leff, 2008; Leinster, 2013; Libbrecht, 2014; Lindlohr, 2017; Louridas, 2016; Macmillan, 1999; Mahon, 2013; Malhotra, 2015; Mason, 2021; Moglia, 2014; Moore, 2015; Nayar, 2020; Osborn, 2015; Phillips, 2018; Quiillin, 2013; Van Rij, 1995; Roman, 2004; Sadideen, 2013; Sapira, 1979; Sartania, 2014; Satiani, 2014; Schreurs, 2020; Šimunovic, 1975; Singh, 2017; Siska, 2015; Smoker, 1984; Stefanidis, 2006; Sun, 2022; Sun, 2021; Sutton, 2018; Swanstrom, 2006; Vu, 1987; West, 2011; White, 2002; Wilson, 2012; Wiseman, 2014; Woodrow, 2022; Zuckerman, 2018 |
| Distinctive | Hayden, 2005 |
| Diversity | Smith, 2012; Tucker, 1965; Woodrow, 2022 |
| Excellence/Excellent | Feng, 2023; Garrud, 2018; Groenier, 2014; Hayden, 2005; Khawar, 2022; Lindlohr, 2017; Meyer, 2019; Moore, 2015; Muller , 2010; Osborn, 2015; Pfeifer, 2017; Sapira, 1979; Satiani, 2014; Sternberg, 2008; Sutton, 2018; Yates, 2013; Zuckerman, 2018 |
| Expertise | Hardre, 2016; Alderson, 2010; Azari, 2019 |
| Gifted | Findeklee, 2019; Gallagher, 2009; Jensen, 2017; Khawar, 2022; Kim, 2012; Moglia, 2014; Moore, 2015; Wolter, 1979 |
| Grit | Burkhart, 2014 |
| Intelligence | Hunter, 1965; Husbands, 2013; Jensen, 2017; Koczwara, 2012; Kulasegaram, 2013; Libbrecht, 2014 |
| Knowledge | Raffoul, 2019; Sapira, 1979 |
| Performance | Berretti, 2018; Burish, 2015; Casey, 2016; Cerutti, 2013; Dabaliz, 2017; Daly, 2014; Dashfield, 2001; Dong, 2013; Dooley, 2021; Edelin, 2001; Edwards, 2013; ElBoghdady, 2021; Gaier, 1952; Glaser, 1954; Groenier, 2014; Harris, 2015; Husbands, 2014; Kim, 2012; Kirby, 1979; Krupat, 2017; Kumwenda, 2018; Libbrecht, 2014; Louridas, 2016; Maan, 2012; Migliaretti, 2017; Peterson, 2005 |
| Professionalism | Van Eaton, 2011; Waheed, 2011 |
| Proficiency | Stefanidis, 2006 |
| Qualified | Althans, 2022 |
| Rock Stars | Lucey, 2010 |
| Skill(s) | Azari, 2019; Bech, 2013; Berner, 2019; Carr, 2009; Dahlin, 2012; Daly, 2014; Dashfield, 2001; Deuchler, 2022; Dooley, 2021; Oliver, 2012; Friedman, 2018; Gallagher, 2009; Gliatto, 2016; Groenier, 2014; Hardre, 2016; Harrington, 2018; Harris, 2015; Hassan, 2007; Hayden, 2005; Hecker, 2009; Henry, 1990; Holmes, 1988; Husbands, 2013; Jensen, 2017; Koenig, 2013; Korets, 2011; Kramp, 2016; Lambe, 2012; Langlois, 2015; Leff, 2008; Leinster, 2013; Krespi, 1986; Lievens, 2012; Lindlohr, 2017; Louridas, 2016; Lucey, 2020; Luursema, 2010; Maan, 2012; Macmillan, 1999; Mahon, 2013; Malhotra, 2015; Mason, 2021; Migliaretti, 2017; Moglia, 2014; Moore, 2015; Muller , 2010; Nayar, 2020; Nothnagle, 2014; Osborn, 2015; Phillips, 2018; Quiillin, 2013; Raffoul, 2019; Van Rij, 1995; Roman, 2004; Rosenthal, 2006; Sapira, 1979; Satiani, 2014; Schropel, 2024; Šimunovic, 1975; Singh, 2017; Siska, 2015; Smoker, 1984; Stefanidis, 2006; Sun, 2022; Sun, 2021; Sutton, 2018; Swanstrom, 2006; Tang, 2014; Vu, 1987; Wanzel, 2002; Watson, 2017; Wenzel, 2016; West, 2011; White, 2002; Woodrow, 2022; Yates, 2013; Yates, 2010; Zuckerman, 2018 |
| Success  or  successful | Burish, 2015; Dabaliz, 2017; Gaier, 1952; Hardre, 2016; Ingersoll, 1965; McPhilemy, 2020; Nazerali-Maitland, 2022; Hardre, 2016; Hewer, 1956; Hunka, 1966; Koenig, 2013; Lievens, 2012; Mendis, 2011; Migliaretti, 2017 |
| The Best | O'Donovan, 2012 |
