# Supplementary material for: Exploring “Talent” in Medical Education: A Scoping Review
Source: Perspect Med Educ. 2026 Feb 4;15(1):75–92. doi: 10.5334/pme.1859 (PMC12879997; doi:10.5334/pme.1859)
Supplement: Appendices. — Appendix A to H. [file pme-15-1-1859-s1.zip › pme-15-1-1859-s1/Appendix_E.docx]

**Table 1: List of included publications that use the word “talent” or “talented”**

| **Year** | **Surname of Lead Author** | **Full Citation** | **Key findings** |
| --- | --- | --- | --- |
| 2016 | Abdulrahman | Abdulrahman M, Makki M, Shaaban S, et al. Specialty preferences and motivating factors: A national survey on medical students from five uae medical schools. Education for Health. 2016;29(3):231. | Medical students in the UAE prioritize intellectual satisfaction, work-life balance, required talent, and a stable future when choosing specialties, with internal medicine, surgery, emergency medicine, and family medicine being the most preferred. Personal interest and flexible working hours are the main reasons for their choices, which can guide policymakers to address specialty shortages in the UAE healthcare system. |
| 2023 | Ackermann | Ackermann J, Baumann J, Pape J. Factors influencing surgical performance and learning progress in minimally invasive surgery - results of an interdisciplinary multicenter study. International Journal of Surgery. 2023;109:2975–2986. | Individual factors such as surgical experience, spatial visualization ability, eye-hand coordination, and age significantly influence surgical performance and learning. Improving and institutionalizing surgical training can help overcome individual learning curves, even for less talented surgeons. |
| 2015 | Aggarwal | Aggarwal R, Swanwick T. Clinical leadership development in postgraduate medical education and training: policy, strategy, and delivery in the UK National Health Service. Journal of Healthcare Leadership. 2015;7:109-122. | The authors advocate for early preparation and formalized pathways to develop management skills in clinicians, emphasizing the need for talent management systems to support junior doctors in the NHS, who are often overlooked due to their transient roles. |
| 1988 | Alexander | Alexander D. Loans for medical students: May exclude talented but poorer students. BMJ. 1988;279; 1561. | A proposed loan scheme for higher education aims to promote economic self-reliance among students but may deter talented students from lower-income backgrounds from pursuing medical education due to the financial burden, potentially impacting career choices and diversity in the medical field. |
| 2011 | Bell | Bell R, Fann S, Morrison J, et al. Determining Personal Talents and Behavioral Styles of Applicants to Surgical Training: A New Look at an Old Problem, Part I. Journal of Surgical Education. 2011:534-541. | Participants (medical school candidates) completed an online survey (the TriMetrix Personal Talent Report) that assessed behavioral style, intrinsic motivators, and dimensional balance. Rankings developed by the program were compared with rankings by the TriMetrix Personal Talent Report. Overall there was little concordance between the two lists. |
| 2012 | Bell | Bell RM, Fann SA, Morrison JE, Lisk JR. Determining Personal Talents and Behavioral Styles of Applicants to Surgical Training: A New Look at an Old Problem, Part II. Journal of Surgical Education 2012;69:23–9. | Identifying applicants' unique behavioral, motivational, and personal talents—beyond traditional application and interview processes—enabled better matching with the program's structure and culture, contributing to their success. |
| 2005 | Boudoulas | Boudoulas H. There is no substitute for talent. Hellenic Journal of Cardiology 2005;46:375. | High-quality physician training hinges on the excellence of both teachers and students, as effective training requires creating talented educators and recruiting outstanding trainees. While curriculums evolve, the ultimate success of education depends on these key factors. |
| 2001 | Carnes | Carnes M, VandenBosche G, Agatisa PK, Hirshfield A, Dan A, Shaver JLF, et al. Using Women’s Health Research to Develop Women Leaders in Academic Health Sciences: The National Centers of Excellence in Women’s Health. Journal of Women’s Health & Gender-Based Medicine 2001;10:39–47. | Despite the substantial increase in women entering U.S. medical schools, their representation in academic leadership remains disproportionately low. Women's health research presents an opportunity to develop talented women physicians and scientists into academic leaders. The National Centers of Excellence in Women's Health aim to address this by integrating leadership plans for women in academic medicine. |
| 2020 | Cobb | Cobb D, Martin TW, Vasilopoulos T, Black EW, Giordano CR. Preparing anesthesiology residents to lead: a leadership seminar. LHS 2019;33:101–11. | The leadership curriculum at the University of Florida significantly improved the leadership skills and values of anesthesiology residents, with those frequently participating in the program perceiving themselves as better equipped to become effective healthcare leaders. This highlights the importance of developing talents through structured leadership training programs. |
| 2022 | Deuchler | Deuchler S, Scholtz J, Ackermann H, Seitz B, Koch F. Implementation of microsurgery simulation in an ophthalmology clerkship in Germany: a prospective, exploratory study. BMC Med Educ 2022;22:599. | Including surgical simulation in the ophthalmology clerkship significantly boosted students' confidence in their microsurgical skills. This training prior to residency helps expose students to surgical fields, identify their talents, and guide their specialty choices. |
| 2021 | Dhaliwal | Dhaliwal G, Hauer KE. Excellence in medical training: developing talent—not sorting it. Perspect Med Educ 2021;10:356–61. | Medical education is shifting from traditional measures of excellence, like grades and test scores, to valuing growth, achievement, and responsiveness to feedback. Teachers are encouraged to adopt a coaching role to develop talent, and schools need to create supportive policies and structures. |
| 2009 | Durso | Durso SC, Christmas C, Kravet SJ, Parsons G, Wright SM. Implications of Academic Medicine’s Failure to Recognize Clinical Excellence. Clinical Medicine & Research 2009;7:127–33. | Failing to recognize clinical excellence in academia can lead to low morale, reduced patient care quality, loss of talented clinicians, lack of commitment to improving care systems, and fewer role models for trainees. Recognizing and valuing clinical excellence is crucial for retaining outstanding clinicians and ensuring high-quality patient care. |
| 2023 | Engel-Rebitzer | Engel-Rebitzer E, Kogan JR, Heath JK. Gender-Based Differences in Language Used by Students to Describe Their Noteworthy Characteristics in Medical Student Performance Evaluations. Academic Medicine 2023;98:844–50. | The study found that gender differences in self-promotion behaviors in the noteworthy characteristics section of medical student performance evaluations  may introduce biases. Men were more likely to use standout and communal words, and discuss scholarship, hobbies, and awards, while women were less likely to highlight these areas, potentially affecting the holistic review of residency applications. |
| 2019 | Findeklee | Findeklee S, Spüntrup E, Radosa JC, Sklavounos P, Hamza A, Solomayer EF, et al. Endoscopic surgery: talent or training? Arch Gynecol Obstet 2019;299:1331–5. | The study found that neither normally talented nor extremely talented junior surgeons could be distinguished based on their knot-tying performance. Factors like musical or sports experience did not significantly impact performance, suggesting that all prospective surgeons can achieve similar results with short interval training. |
| 2019 | Friedman | Friedman AH. Talent alone is not enough: lessons learned in a decade as chief. Current Opinion in Cardiology 2019;34:87–93. | The review highlights that a talented physician must go beyond scientific knowledge and technical skills, incorporating a commitment to patient safety, well-being of themselves and colleagues, and a culture of professional behavior. It also emphasizes the importance of sleep, quality, safety, professionalism, and communication in clinical practice. |
| 2016 | Hardre | Hardré PL, Nihira M, LeClaire E, Moen M. Defining Expertise in Gynecologic Surgery: Perspectives of Expert Gynecologic Surgeons. Female Pelvic Med Reconstr Surg. 2016;22(6):399-403. | Surgical expertise is a complex concept that can be described using themes such as knowledge, technical skills, clinical experience, adaptability, continuous learning, communication and professional recognition. Continuous learning and challenge-seeking are vital to maintaining expertise, while effective communication contributes to the recognition of expertise by colleagues. |
| 2005 | Hayden | Hayden SR, Hayden M, Gamst A. What characteristics of applicants to emergency medicine residency programs predict future success as an emergency medicine resident? Acad Emerg Med. 2005;12(3):206–10. | Medical school attended was one of the most consistent and strongest predictors of success in an emergency medicine residency. Distinctive factors, such as being a top-level athlete, musician or student leader were another key predictor of success. |
| 2017 | Jensen | Jensen RD, Christensen MK, LaDonna KA, Seyer-Hansen M, Cristancho S. How Surgeons Conceptualize Talent: A Qualitative Study Using Sport Science as a Lens. J Surg Educ. 2017;74(6):992–1000. | Surgical talent is shaped by a combination of individual skills, a mix of competencies and a nurturing environment. There was discordance among surgeons on whether surgical talent was based solely on individual abilities or if it was a result of an interaction between the individual and their environment. |
| 2022 | Khawar | Khawar A, Frederiks F, Nasori M, Mak M, Visser M, van Etten-Jamaludin F, Diemers A, Van Dijk N. What are the characteristics of excellent physicians and residents in the clinical workplace? A systematic review. BMJ Open. 2022;12(9):e065333. | There were three key clusters that defined excellence in medical doctors: competence, motivation, and personality. These findings aligned with Renzulli’s model of gifted behaviour. |
| 2012 | Kim | Kim KJ, Kee C. Gifted students' academic performance in medical school: a study of Olympiad winners. Teach Learn Med. 2012;24(2):128–32. | Students with exceptional talent in science and mathematics outperformed their peers in total GPA; however, the performance gap diminished over time, with no difference in clinical clerkship performance or national licensing exam (KMLE) scores. |
| 2016 | Kramp | Kramp KH, van Det MJ, Hoff C, et al. The predictive value of aptitude assessment in laparoscopic surgery: a meta-analysis. Med Educ. 2016;50(4):409–27. | Performance on aptitude assessments, specifically simulation-based assessments were associated with laparoscopic skill level. |
| 2017 | Lindlohr | Lindlohr C, Lefering R, Saad S, et al. Training or non-surgical factors-what determines a good surgical performance? A randomised controlled trial. Langenbecks Arch Surg. 2017;402(4):645–653. | The type of teaching method used during laparoscopic training was the most influential factor in enhancing learning success. Multimedia training significantly increased surgical performance. |
| 2010 | Lucey | Lucey CR, Sedmak D, Notestine M, Souba W. Rock stars in academic medicine. Acad Med. 2010;85(8):1269–75. | “Rock star” faculty members brought significant value by enhancing institutional reputation through exceptional research or clinical work. These “rock stars” often required substantial resources and autonomy, and the retention of such individuals was challenging as they frequently considered offers from other prestigious institutions. |
| 2020 | Lucey | Lucey CR, Saguil A. The Consequences of Structural Racism on MCAT Scores and Medical School Admissions: The Past Is Prologue. Acad Med. 2020;95(3):351–356. | Differences in MCAT scores between groups well represented in medicine and groups underrepresented in medicine likely stem from disparities in opportunities caused by structural racism. This opportunity gap might be addressed through changes in the medical school admission process, pipeline programs and education of physician-citizens. |
| 2016 | Meier | Meier M, Horton K, John H. Da Vinci Skills Simulator: is an early selection of talented console surgeons possible? J Robot Surg. 2016;10(4):289–296. | Performance in virtual simulator modules was dependent on prior experience with robotic and laparoscopic surgery and age. These factors did not impact the learning curves of proband groups. |
| 2014 | Moglia | Moglia A, Ferrari V, Morelli L, et al. Distribution of innate ability for surgery amongst medical students assessed by an advanced virtual reality surgical simulator. Surg Endosc. 2014;28(6):1830–7. | Two distinct subpopulations of medical students were identified based on their innate aptitude for manipulative and psychomotor skills required for surgery. A small group outperformed their peers, while a larger group performed significantly worse than their peers. There was no influence of video game performance on skills simulator performance. |
| 2014 | Nothnagle | Nothnagle M, Radlinski H, Magee SR, et al. Developing future leaders in reproductive health through a scholarly concentration for medical students. Contraception. 2014;90(5):508–13. | Scholarly concentration programs contributed to the knowledge, skills and attitudes of students through longitudinal mentoring, independent project work and paper support. These programs might be used to prepare talented individuals to become future leaders. |
| 2017 | Pfeifer | Pfeifer CM, Bourm KS. Diagnostic Radiology Continues to Attract Talented Applicants. J Am Coll Radiol. 2017;14(4):545–546. | Data from the 2015-2016 residency recruitment season showed an increase in new graduates from US medical schools selecting the specialty of diagnostic radiology. This specialty remained competitive and continued to attract strong applicants despite prior concerns about declining interest. |
| 2017 | Porter | Porter SE, Razi AE, Ramsey TB. Novel Strategies to Improve Resident Selection by Improving Cultural Fit. J Bone Joint Surg Am. 2017;99(22):e120. | Orthopaedic residency selection might be improved by addressing reliance on metrics like USMLE scores and adoption of standardized letters of recommendation. Strong program brand development and evaluation of cultural fit might ensure better alignment between residents and program values. |
| 2018 | Preece | Preece R, Ben-David E, Rasul S, et al. Are we losing future talent? A national survey of UK medical student interest and perceptions of cardiothoracic surgery. Interact Cardiovasc Thorac Surg. 2018;27(4): 525–529. | UK medical students with an interest in surgery remain keen on pursuing a career in cardiothoracic surgery, but their enthusiasm declines over the course of their undergraduate studies. This was linked to limited exposure to the specialty in both curriculum and extracurricular activities. |
| 2023 | Rathmell | Rathmell WK. Learning to pivot:  Developing talent and a championship team. Trans Am Clin Climatol Assoc. 2023;133: 81–92. | Challenges in cultivating physician-scientists include long training periods, financial sacrifice and rigid career pathways that limit flexibility. The pipeline must be expanded and diversified to address these issues. A mentorship-driven approach might best develop talent and meet the growing demand for skilled physician-scientists. |
| 2004 | Roman | Roman SA. Addressing the urban pipeline challenge for the physician workforce: the Sophie Davis model. Acad Med. 2004;79(12): 1175–83. | The Sophie Davis model is a combined BS/MD program that supports talented inner-city youths to increase diversity in medical education, provide improved academic success and address the projected physician shortage in the United States. |
| 2006 | Rosenthal | Rosenthal R, Gantert WA, Scheidegger D, et al. Can skills assessment on a virtual reality trainer predict a surgical trainee's talent in laparoscopic surgery? Surg Endosc. 2006;20(8): 1286–90. | Medical students without prior laparoscopic experience could successfully perform tasks on the Xitact LS 500 surgical simulator. Performance measurements followed predictable logarithmic learning curves, suggesting innate ability plays a role in laparoscopic proficiency. |
| 2013 | Sadideen | Sadideen H, Alvand A, Saadeddin M, Kneebone R. Surgical experts: born or made? Int J Surg. 2013;11(9): 773–8. | Innate talent plays a role in surgical expertise but deliberate practice is essential for skill acquisition. Surgical expertise requires both psychomotor and non-technical skills, and an expertise-based approach in training programs could help ensure high-quality surgical education. |
| 2014 | Satiani | Satiani B, Sena J, Ruberg R, et al. Talent management and physician leadership training is essential for preparing tomorrow's physician leaders. J Vasc Surg. 2014;59(2): 542–6. | Physicians bring unique advantages to leadership roles; however, they also face challenges due to a lack of formal administrative training. The Talent Management and Leader Development Academy was developed to equip physicians with leadership, financial and strategic skills. The program received positive feedback, though concerns about attendance, workload balance and group collaboration were raised. |
| 2008 | Sternberg | Sternberg RJ. Assessing students for medical school admissions: is it time for a new approach? Acad Med. 2008;83(10 Suppl):S105–10. | The Theory of Successful Intelligence, which emphasizes analytical, creative and practical abilities as key predictors of success may have the potential to better predict the success of medical school applicants than standardized tests such as the SAT and MCAT. |
| 2015 | Subramaniam | Subramaniam A, Silong AD, Uli J, et al. Effects of coaching supervision, mentoring supervision and abusive supervision on talent development among trainee doctors in public hospitals: moderating role of clinical learning environment. BMC Med Educ. 2015;15:129. | Mentoring and coaching were positively associated with talent development, while abusive supervision and talent development had no association. The clinical learning environment moderated the relationship between the trainee and supervisor, resulting in the strengthening or weakening of different supervisory styles. |
| 2021 | Sun | Sun R, Wang Y, Fast A. Influence of musical background on surgical skills acquisition. Surgery. 2021;170:175-80. | The study found that a musical background is associated with better performance in fundamental surgical skills among novices, particularly in technique quality. This suggests that musicality may be a useful marker for identifying candidates with potential for surgical training. |
| 2018 | Sutton | Sutton PA, Beamish AJ, Rashid S. Attributes of excellent surgical trainers: An analysis of outstanding trainers. International Journal of Surgery. 2018;52:371-375. | Talented surgical trainers are essential for teaching, mentoring, and inspiring trainee surgeons, with the best trainers being recognized for their ability to combine excellent patient care with effective training. The Silver Scalpel Award highlights these exceptional trainers who go above and beyond for their trainees, emphasizing the importance of supporting and celebrating their contributions. |
| 1965 | Tucker | Tucker M. The Role of the Women's Auxiliary to the National Medical Association in the Talent Recruitment Program. Journal of the National Medical Association. 1965;57(6):453-4. | The Women’s Auxiliary to the National Medical Association recognizes the importance of scholarships and grants to attract students and the need for a concerted effort to integrate the talent recruitment program with other anti-poverty initiatives. |
| 2016 | Wenzel | Wenzel V, Gravenstein N. Anesthesiology Mentoring. Current Opinion in Anesthesiology. 2016;29(6): 698-702. | Mentoring is crucial for identifying and developing latent talent among medical students and residents, guiding them through their careers, and ensuring the success of the entire department. Effective mentoring involves creating professional plans, overcoming challenges, and providing role models to help mentees avoid learning lessons the hard way. |
| 2002 | White | White A. Resident Selection: Are we putting the cart before the horse?  Clinical Orthopaedics and Related Research. 2002;399: 255–259. | The selection of orthopaedic residents should prioritize societal goals and responsibilities, aiming to produce graduates with talents in diversity, culturally competent care, research, leadership, administration, and education. Traditional selection criteria, such as grades and test scores, have limitations and biases, necessitating a substantial change in the selection process to achieve significant societal goals. |
| 2022 | Woodrow | Plonsker J, Benzil D, Air E, et al. Gender Equality in Neurosurgery and Strategic Goals Toward a More Balanced Workforce. Neurosurgery. 2022;90:642–647. | The Women in Neurosurgery (WINS) and the American Association of Neurological Surgeons emphasize the need to recruit and retain diverse talent in neurosurgery by providing early mentorship, enforcing parental leave policies, ensuring compensation equity, and establishing zero-tolerance policies for harassment. They aim for women to comprise 30% of neurosurgery residents by 2030 and practicing neurosurgeons by 2038. |
| 2018 | Zuckerman | Zuckerman S, Kelly P, Dewan M, et al. Predicting Resident Performance from Preresidency Factors: A Systematic Review and Applicability to Neurosurgical Training. World Neurosurg. 2018; 110:475-484. | Athletic or musical talent was found to moderately correlate with faculty evaluations of neurosurgical residents, suggesting that these talents may be considered as part of the selection process for predicting resident success. However, the overall ability to predict success using these factors remains limited. |
