# Supplementary material for: Exploring “Talent” in Medical Education: A Scoping Review
Source: Perspect Med Educ. 2026 Feb 4;15(1):75–92. doi: 10.5334/pme.1859 (PMC12879997; doi:10.5334/pme.1859)
Supplement: Appendices. — Appendix A to H. [file pme-15-1-1859-s1.zip › pme-15-1-1859-s1/Appendix_F.docx]

**Appendix F: Glossary of synonymous terms found in Preyra et al. Scoping Review on Exploring “Talent” in Medical Education.**

*Please note that the term “talent” is missing from this table. The definitions of “talent” for this study are listed in Table 2 of the paper.*

| **Term analogous to the term Talent** | **Definition** |
| --- | --- |
| Ability | The innate capacity to perform specific psychomotor, cognitive, or interpersonal tasks, regarded as immutable through practice or experience and foundational to the execution of a broad range of skills. |
| Accomplishment | A concrete, observable outcome that reflects successful completion or a goal or task. |
| Achievement or Attainment | The measurable realization of set objectives or benchmarks that signify progress or excellence in training or practice. |
| Aptitude | A natural propensity for learning or performance in a specific domain which influences how quickly and effectively a person can develop skills. |
| Attributes | Descriptive characteristics or personal qualities that influence how an individual approaches learning and professional challenges, but do not by themselves determine performance. |
| Best | The highest level of performance, achievement, or recognition relative to peers. |
| Competency | A demonstrable and measurable integration of knowledge, skills, attitudes, and behaviours necessary for effective professional performance, typically aligned with formal frameworks (e.g. CanMEDS or ACGME milestones). |
| Excellent | A high standard of performance, often exceeding expectations. |
| Expert/Expertise | Individuals who demonstrate consistent high-level performance, extensive knowledge, and refined skills in a specific domain developed over sustained deliberate practice. |
| Gifted | Exceptional innate capabilities in cognitive, creative, or psychomotor domains, often emerging early in life and independently of formal instruction. |
| Grit | An individual’s sustained passion, effort, and perseverance toward long term goals, despite challenges and setbacks. |
| Intelligence | The cognitive capacity to acquire, process and apply information to different contexts. |
| Knowledge | The organized and contextualized body of facts, concepts, and theoretical frameworks internalized through education, experience, or reflection. |
| Performance | The observable execution of tasks in a given setting, typically evaluated through standardized assessments or clinical metrics. |
| Proficiency | A level of skill or accuracy in a specific domain that meets or exceeds the standard required for effective and reliable performance. |
| Qualified | An individual who has met predefined criteria, such as licensure, certification, or completion of training necessary to engage in a specific role or scope of practice. |
| Rock star | An individual perceived as standout performers or influential figure in academic or clinical settings, often associated with charisma, ambition, and institutional visibility rather than competence alone. |
| Skills | The learned capacities to perform specific tasks that are developed through deliberate practice and dependent on the integration of multiple abilities. |
| Successful | Individuals who achieve targeted outcomes, as evidenced by objective measures of effectiveness, efficiency, or recognition by peers. |
