# Supplementary material for: Exploring “Talent” in Medical Education: A Scoping Review
Source: Perspect Med Educ. 2026 Feb 4;15(1):75–92. doi: 10.5334/pme.1859 (PMC12879997; doi:10.5334/pme.1859)
Supplement: Appendices. — Appendix A to H. [file pme-15-1-1859-s1.zip › pme-15-1-1859-s1/Appendix_G.pptx]

## Slide 1
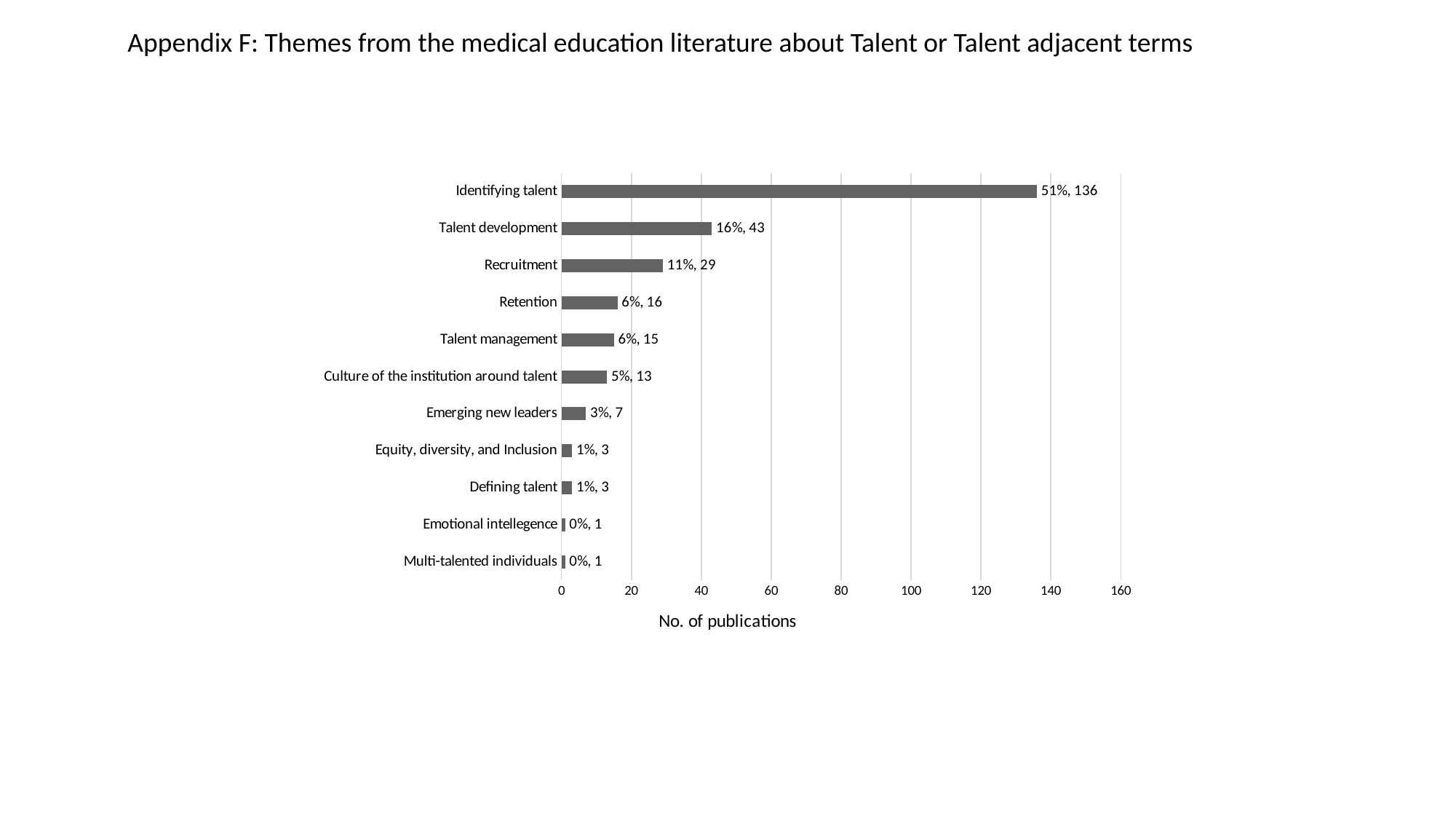

Appendix F: Themes from the medical education literature about Talent or Talent adjacent terms
### Chart
| Category | Freq |
|---|---|
| Multi-talented individuals | 1.0 |
| Emotional intellegence | 1.0 |
| Defining talent | 3.0 |
| Equity, diversity, and Inclusion | 3.0 |
| Emerging new leaders | 7.0 |
| Culture of the institution around talent | 13.0 |
| Talent management | 15.0 |
| Retention | 16.0 |
| Recruitment | 29.0 |
| Talent development | 43.0 |
| Identifying talent | 136.0 |
