# Supplementary material for: Exploring “Talent” in Medical Education: A Scoping Review
Source: Perspect Med Educ. 2026 Feb 4;15(1):75–92. doi: 10.5334/pme.1859 (PMC12879997; doi:10.5334/pme.1859)
Supplement: Appendices. — Appendix A to H. [file pme-15-1-1859-s1.zip › pme-15-1-1859-s1/Appendix_H.docx]

**Theories and Frameworks for Perceptual Talent**

| **Last name of first author** | **Year of Publication** | **Framework for…** | **Framework Details** |
| --- | --- | --- | --- |
| Harrington | 2018 | Fundamental characteristics | Visuospatial and perceptual aptitudes as defined by PicSOr cube comparison & card rotations |
| Hassan | 2007 | Spatial perception | Lameris TNO test and Stumpf–Fay Cube Perspectives Test |
| Krupat | 2017 | Visuospatial skills | Various visuospatial & psychomotor skills testing |
| Krespi | 1986 | Surgical aptitude | Perceptual ability test (2D & 3D); Manual Dexterity test (Dexterity loop, mirror test, microscope test) |
| Luursema | 2010 | Visuo-spatial ability (in colonoscopy) | Carroll identifies five main third-tier factors nested within visuo-spatial ability, namely Visualization, Spatial relations, Speed of closure, Flexibility of closure, and Perceptual speed. |
| Maan | 2012 | Predictors of surgical performance | Visual-spatial perception; Psychomotor aptitude |
| Mason | 2021 | Innate arthroscopic and laparoscopic surgical skills | Current literature suggests that video game experience/frequency, psychomotor and visuospatial aptitude, and perceptual ability are among the most promising predictive indicators of baseline simulator performance. |

**Theories and Frameworks for Technical Talent**

| **Last name of first author** | **Year of publication** | **Framework for…** | **Framework Details** |
| --- | --- | --- | --- |
| Kirby | 1979 | Dexterity | Dexterity tests |
| Lindlohr | 2017 | Good surgical performance | Surgical performance was then evaluated using a modified objective structured assessment of technical skills (OSATS). Participants were classified as ‘Skilled’ (high score in the pre-test), ‘Good Learner’ (increase from pre- to post-test) or ‘Others’ based on the OSATS results. |
| Louridas | 2016 | Surgical technical skills | Learning curves (LCs) of both open and laparoscopic simulated technical skills. |
| Macmillan | 1999 | Innate ability and skills for endoscopic manipulations | Advanced Dundee Endoscopic Psychomotor Tester (ADEPT) |
| McClusky | 2005 | Surgical skills | Laparoscopic virtual reality simulator |
| Moglia | 2014 | Innate surgical ability in medical students | da Vinci Skills Simulator (manipulative & psychomotor skills) |
| Moore | 2015 | Otolaryngology resident performance | Surgical aptitude test as a predictor |
| Wolter | 1979 | Gifted hands (for ocular surgery) | Dexterity test |

**Theories and Frameworks for Non-Technical Talent**

| **Last name of first author** | **Year of publication** | **Framework for…** | **Framework Details** |
| --- | --- | --- | --- |
| Aggarwal | 2015 | Leadership competency | Medical Leadership Competency Framework (MLCF) |
| Alderson | 2010 | Competence | Howell's model of the development of cognisance and competence (Howell 1982) |
| Azari | 2019 | Expertise | TEMPEST model describing the general framework of expertise (Adapted from Feltovich, Ford and Hoffmann, 1997). |
| Bell | 2011 | Personal talents and behavioural styles | The Success Insights Wheel graphically compares applicants with the job benchmark. |
| Burkhart | 2014 | Grit | Measured grit using a Likert scale |
| Carr | 2009 | Emotional intelligence | Four-branch model of emotional intelligence (Mayer & Salovey 1997). |
| Engel-Rebitzer | 2023 | Noteworthy characteristics | Sets forth some word categories: ability words, standout words, grindstone words, communal words, agentic words |
| Feng | 2023 | Excellent pediatric residents | 6 categories: professional spirit, clinical skills, communication ability, learning ability, mental capacity and research ability |
| Hayden | 2005 | Predictions of future success | Using regression modeling, it may be possible to predict future resident performance from characteristics contained in residency applications. |
| Henry | 1990 | Academic performance | The independent variable was the Developing Cognitive Abilities Test which participants took as part of a battery of MEDPREP admissions tests. The test measured the characteristics and abilities that contribute to academic performance. |
| Holmes | 1988 | Fitness and aptitude to practice medicine | The Critical Incident Technique (CIT) used in this study elicited 484 behaviours from which categories, subcatego- ries and objectives were developed to describe the essential behaviours of aptitude and fitness. |
| Libbrecht | 2014 | Emotional intelligence | EI may predict performance on the interpersonal aspects of medical work. |
| Malhotra | 2015 | Personality dimensions | Movement self-consciousness (MS-C) - the propensity to consciously monitor movements; Conscious motor processing (CMP) - the propensity to consciously control movements |
| Matarazzo | 1972 | Intellectual caliber (of med students) | IQ tests, MCAT, college grades, attrition rates |
| Mathers | 2016 | Selection of med school applicants | UK Clinical Aptitude Test (UKCAT) |
| Mathew | 2018 | Medical aptitude | Various standardized tests (e.g. MCAT, UKCAT) |

| McGaghie | 2002 | Readiness for Medical Education | MCAT history |
| --- | --- | --- | --- |
| McManus | 2013 | First year outcome (in medical school) | UKCAT aptitude test |
| McPhilemy | 2020 | Orthopaedic surgery candidate selection | Personality assessments, emotional intelligence assessments |
| Mercer | 2018 | Medical student selection | UMAT & GAMSAT |
| Peterson | 2005 | Performance | Medical Gross Anatomy as a Predictor of Performance on the USMLE Step 1 |
| Phillips | 2018 | Resident performance (in surgery) | Five Factor Model of personality characteristics: agreeableness, conscientiousness, extroversion, neuroticism, and openness |
| Pounds | 2017 | Empathic communication skills | Various written and oral tests developed by the authors. |
| Quiillin | 2013 | Success in surgical residency | Kolb Learning Style Inventory (LSI) |
| Schreurs | 2020 | Selection (of med school students) | Downing's Validity Framework: content, response process, internal structure, relationship to other variables, consequences |
| Sobral | 1995 | Diagnostic ability (in med students) | Diagnostic Thinking Inventory (DTI) |
| Subramaniam | 2015 | Talent development | Structural model for talent development: includes coaching and mentoring |
| Sutton | 2018 | Excellent surgical trainers | Key attributes: Leadership skills, Resourcefulness, Training and Development, Professionalism, Communication Skills |
| Toale | 2024 | Competent, independent surgeons | Selection metrics and performance outcomes: surgical aptitude, clinical judgment, interpersonal skills, professional development, suitability for specialist training |
| Wiseman | 2014 | Leadership practice (in med school) | Five Disciplines of Multipliers: The Empire Builder, The Tyrant, The Know-It-All, The Decision Maker, The Micromanager |
